# Tmod1 and CP49 Synergize to Control the Fiber Cell Geometry, Transparency, and Mechanical Stiffness of the Mouse Lens

**DOI:** 10.1371/journal.pone.0048734

**Published:** 2012-11-07

**Authors:** David S. Gokhin, Roberta B. Nowak, Nancy E. Kim, Ernest E. Arnett, Albert C. Chen, Robert L. Sah, John I. Clark, Velia M. Fowler

**Affiliations:** 1 Department of Cell Biology, The Scripps Research Institute, La Jolla, California, United States of America; 2 Department of Biological Structure, University of Washington, Seattle, Washington, United States of America; 3 Department of Bioengineering, University of California San Diego, La Jolla, California, United States of America; University of Missouri-Columbia, United States of America

## Abstract

The basis for mammalian lens fiber cell organization, transparency, and biomechanical properties has contributions from two specialized cytoskeletal systems: the spectrin-actin membrane skeleton and beaded filament cytoskeleton. The spectrin-actin membrane skeleton predominantly consists of α_2_β_2_-spectrin strands interconnecting short, tropomyosin-coated actin filaments, which are stabilized by pointed-end capping by tropomodulin 1 (Tmod1) and structurally disrupted in the absence of Tmod1. The beaded filament cytoskeleton consists of the intermediate filament proteins CP49 and filensin, which require CP49 for assembly and contribute to lens transparency and biomechanics. To assess the simultaneous physiological contributions of these cytoskeletal networks and uncover potential functional synergy between them, we subjected lenses from mice lacking Tmod1, CP49, or both to a battery of structural and physiological assays to analyze fiber cell disorder, light scattering, and compressive biomechanical properties. Findings show that deletion of Tmod1 and/or CP49 increases lens fiber cell disorder and light scattering while impairing compressive load-bearing, with the double mutant exhibiting a distinct phenotype compared to either single mutant. Moreover, Tmod1 is in a protein complex with CP49 and filensin, indicating that the spectrin-actin network and beaded filament cytoskeleton are biochemically linked. These experiments reveal that the spectrin-actin membrane skeleton and beaded filament cytoskeleton establish a novel functional synergy critical for regulating lens fiber cell geometry, transparency, and mechanical stiffness.

## Introduction

The ocular lens consists of successive layers of hexagonally packed fiber cells, whose structural properties provide lens transparency [1]. The hexagonally packed three-dimensional architecture of lens fiber cells arises during the complex morphogenetic program of fiber cell differentiation, in which the short cuboidal epithelial cells along the lens equator align into meridional rows and begin to elongate [2], [3]. As the posterior-most cell in each meridional row differentiates into a lens fiber cell, it begins to express the lens-specific gene expression program and continues to elongate until its apical and basal ends terminate at the poles of the lens. During lens growth, nascent cortical fiber cells are deposited on top of older elongating fiber cells, forming concentric shells of hexagonally packed and radially aligned fiber cells. As cells move inward and mature in the deep cortex, these aging cells degrade their nuclei and intracellular organelles to enhance their optical clarity [4], [5], [6]. The lens fiber cells remain radially aligned and hexagonally packed throughout differentiation in the cortex, with their membranes developing increasingly elaborate morphological protrusions to form large paddle-like structures in the deep cortex, which are then remodeled into smoother membrane contours in the organelle-free fiber cells of the lens nucleus [6], [7], [8], [9], [10]. This stereotypic growth process is believed to be important for establishing the biomechanical properties of the mature lens, which, during focusing and accommodation, withstands frequent mechanical loading imposed by the ciliary muscle and transmitted to the lens via the ciliary zonule [11].

A key regulator of lens fiber cell architecture and mechanical properties is a specialized intermediate filament cytoskeleton consisting of two fiber cell-specific intermediate filament proteins, CP49 (phakinin) and filensin, that coassemble into structures known as beaded filaments [12]. CP49 and filensin are expressed upon initiation of fiber cell differentiation, predominantly localizing to the fiber cell membrane in young fiber cells in the shallow cortex, and are proteolytically processed and become more cytoplasmic as the cells age and lose their organelles [13], [14], [15]. CP49 and filensin assembly into beaded filaments is mutually codependent, with genetic deletion of either one resulting in reduced levels of the other, thus eliminating all beaded filaments in the lens [16], [17], [18], [19], [20]. Targeted deletion of CP49 or filensin does not affect fiber cell differentiation in the outer cortex, including radial cell alignment and formation of membrane protrusions, but the maturing fiber cells in the inner cortex display striking morphological abnormalities, failing to maintain their paddle-like membrane protrusions and becoming grossly misaligned [16], [17], [19], [21]. The importance of beaded filaments in regulating the mechanical properties of the lens has been demonstrated via biomechanical testing of CP49-null lenses, which, when subjected to ramp compression and decompression cycles, exhibit decreased stiffness and slightly increased resilience compared to wild-type lenses [22]. Furthermore, evidence has hinted at a potentially intriguing relationship between tissue mechanical properties and maintenance of transparency during lens development and aging. For example, CP49 or filensin deletion leads to subtle, age-dependent opacification and loss of optical quality in mice, as detected by slit-lamp examination and laser ray tracing [16], [17], [19], while *Bfsp2/CP49* gene mutations lead to hereditary cataracts in humans [23], [24], [25], [26]. Moreover, the concentrations of CP49 and filensin in the lens cortex decrease during opacification in a rat model of hereditary cataract [27].

A second key cytoskeletal regulator of lens fiber cell architecture is the spectrin-actin membrane skeleton. The lens membrane skeleton consists of actin filaments, which are crosslinked by α_2_β_2_-spectrin strands, stabilized along their sides by γ-tropomyosin (γTM), and capped at their barbed and pointed ends by adducin and tropomodulin 1 (Tmod1), respectively [28], [29], [30], [31]. The entire spectrin-actin network is then tethered to the fiber cell membrane via spectrin’s interaction with ankyrin-B [32], [33], which, in turn, is linked to the adhesion receptor NrCAM [33] and possibly N-cadherin [34]. The biological significance of the lens membrane skeleton has been probed via genetic deletion of Tmod1, which leads to F-actin disassembly, reduced levels of γTM, and widespread disruption of the spectrin-actin lattice in differentiating fiber cells, but with no apparent adverse effects on lens transparency [35]. Fiber cells in Tmod1-null lenses are characterized by abnormal membrane protrusions and cell shapes and disordered packing geometry, highlighting the importance of the membrane skeleton in directing fiber cell architecture during lens development [35]. While the spectrin-actin network is disrupted in all Tmod1-null fiber cells, fiber cell disorder manifests in an unexpectedly patchy manner in the outer lens cortex, with small disordered patches that are first observed in elongating fiber cells and subsequently increase in size (but not number) as fiber cells continue to differentiate in the cortex [36]. This has motivated a model in which a nonuniform stress distribution in the lens, along with an abnormally low tolerance to mechanical loading due to the disrupted and irregular spectrin-actin network, results in localized foci of tissue damage that gradually enlarge over time [36].

Surprisingly, the relationship between the spectrin-actin membrane skeleton and cell mechanics has only been explicitly studied in the red blood cell (RBC) membrane, in which the membrane skeleton was originally discovered. In RBCs, defects in membrane skeleton proteins lead to pathological alterations in membrane extensibility and stiffness [37], [38]. In the case of Tmod1 ablation, resultant spectrin-actin network disruption leads to mechanically compromised cell membranes in both primitive erythroid cells as well as definitive adult RBCs, as determined by micropipette aspiration, osmotic fragility, and ektacytometry experiments [39], [40]. While spectrin-actin network disruption and membrane integrity defects in Tmod1-null RBCs are indeed reminiscent of the network disruption, membrane destabilization, and cellular disorder phenotypes observed in Tmod1-null lenses [35], [36], direct experimental evidence relating the membrane skeleton to the mechanical properties of the lens has remained elusive. The paucity of studies on the effects of membrane skeleton defects on lens mechanics is surprising, as the regular geometry and stratified architecture of the lens facilitate tissue-scale measurements of intact lenses as well as identification of depth-dependent mechanical heterogeneities.

While both F-actin and intermediate filaments are essential regulators of cell morphology and mechanics in virtually all metazoan cells, the relative contributions of the spectrin-actin network and beaded filaments in regulating three-dimensional hexagonal fiber cell packing and lens mechanics are poorly understood. Notably, the Tmod1-null mouse used in previous lens fiber cell architecture studies was generated on a mixed FvBN/129SvEv/C57Bl6 background with an endogenous mutation in CP49 that results in an absence of beaded filaments [18], [20], [35], [36], [41]. While beaded filaments clearly regulate lens fiber cell membrane morphology and cell alignment during maturation in the deep cortex and lens nucleus [19], [21], beaded filaments may also have a more subtle, as-yet-undiscovered function in regulating fiber cell morphology in the elongating and differentiating fiber cells of the shallow cortex, whose importance could be potentiated in the absence of Tmod1 and a disrupted spectrin-actin network. Hence, the possibility exists that the initial formation and subsequent expansion of disordered fiber cell patches in the shallow cortex of Tmod1-null lenses may have actually been due to the combined absence of Tmod1 and beaded filaments. Therefore, in this study, we dissected the distinct but overlapping contributions of the spectrin-actin membrane skeleton and beaded filament cytoskeleton to lens fiber cell organization, transparency, and biomechanics. Toward this end, the *in vivo* structural, optical, and mechanical roles of the spectrin-actin network and beaded filament cytoskeleton were analyzed via phenotypic analysis of *Tmod1+/+* and *Tmod1−/−* lenses from mice on both *CP49+/+* and *CP49−/−* backgrounds. Our findings show that deletion of both Tmod1 and CP49 results in defects in lens fiber cell organization, light scattering, and compressive mechanical properties that are distinct from defects arising from deletion of either Tmod1 or CP49 alone. These data, in combination with the biochemical finding that Tmod1, actin, CP49, and filensin coexist in a protein complex, indicate that the spectrin-actin network and beaded filament cytoskeleton are both functionally and biochemically coupled, thereby highlighting a novel functional cytoskeletal synergy important for mammalian lens physiology.

## Materials and Methods

### Ethics Statement

All procedures were performed in strict accordance with the recommendations in the Guide for the Care and Use of Laboratory Animals by the National Institutes of Health and enforced by the Institutional Animal Care and Use Committee (IACUC) at The Scripps Research Institute (TSRI). Animals were housed with free access to food and water, with a 12∶12 h light:dark cycle, and sacrificed by halothane inhalation followed by cervical dislocation. Animals were between 1 and 16 mo old at the time of sacrifice. These procedures were approved by the IACUC at TSRI on October 6, 2011 (protocol number 08-0087-2), and all efforts were made to minimize animal suffering.

### Mouse Strains and Genotyping

**Figure 1:**
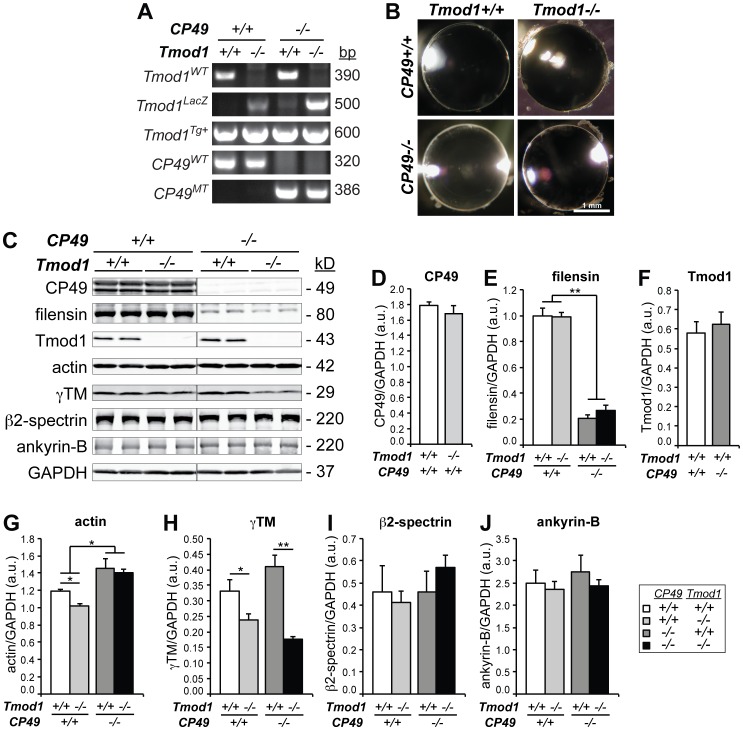
Cytoskeletal protein levels in mouse lenses lacking Tmod1 and/or CP49. (A) PCR genotyping of *Tmod1^WT^*, *Tmod1^LacZ^*, *Tmod1^Tg+^*, *CP49^WT^*, and *CP49^MT^* alleles in *Tmod1+/+* and *Tmod1−/−* lenses in both *CP49+/+* and *CP49−/−* backgrounds. (B) Normal anatomy and absence of overt cataracts in 1-4-month-old dissected lenses lacking Tmod1 and/or CP49. (C) Western blots for CP49, filensin, Tmod1, actin, γTM, β2-spectrin, and ankyrin-B in 2-mo-old lenses lacking Tmod1 and/or CP49. GAPDH was used for normalization, with GAPDH probed on each cytoskeletal protein blot. Each lane was loaded with a lens gel sample from an individual mouse. (D-J) Western blot band intensities of (D) CP49, (E) filensin, (F) Tmod1, (G) actin, (H) γTM, (I) β2-spectrin, and (J) ankyrin-B were densitometrically quantified with normalization to GAPDH. Error bars reflect mean±SEM of *n* = 3 lanes/genotype within a single blot. *, *p*<0.05; **, *p*<0.01.

*Tmod1−/−^Tg+^* mice were originally created on a mixed FvBN/129SvEv/C57Bl6 background and have been described previously [35], [36], [40], [42], [43]. In this strain, the embryonic lethality of the *Tmod1−/−* mouse [44] was rescued by a Tmod1-overexpressing transgene under the control of the cardiac-specific α-myosin heavy chain (α*MHC*) promoter (*Tg(*α*MHC-Tmod1*), referred to as *Tg+* in the text) [42]. Thus, *Tmod1+/+^Tg+^* animals expressing the α*MHC* transgene in their heart had normal levels of Tmod1 in all tissues except the heart, while *Tmod1−/−^Tg+^* animals had no Tmod1 in any tissues except the heart [42]. Due to an endogenous mutation in the *Bfsp2/CP49* gene present in the FvBN strain [20], the lenses in these mixed-background *Tmod1−/−^Tg+^* mice lack beaded filament protein CP49 (*CP49−/−*) and have reduced levels of filensin [35]. Restoration of the wild-type *Bfsp2/CP49* allele (*CP49+/+*) was achieved by twice backcrossing *Tmod1+/−^Tg+^* mice with C57Bl6 mice and screening for *Tmod1+/−^ Tg+^* progeny containing the wild-type and targeted *Tmod1^lacZ^* alleles, the α*MHC-Tmod1* transgene, and wild-type (but not mutant) *Bfsp2/CP49* alleles (Fig. 1A). Primers for genotyping of wild-type and targeted *Tmod1^lacZ^* alleles and the α*MHC-Tmod1* transgene were as in [42]. Primers for genotyping of wild-type and mutant *Bfsp2/CP49* alleles were as in [20].

### Preparation of Lens Extracts and Western Blotting

Lenses from 1- to 3-mo-old mice were dissected in warmed Ca^2+^-Mg^2+^-free Dulbecco’s PBS (DPBS) and decapsulated. The 2 lenses from each mouse were placed in one tube containing 100 µl of SDS sample buffer, vigorously vortexed, and boiled for 5 min. The SDS-solubilized proteins were transferred to a new tube, leaving the rigid nucleus behind. Proteins were separated on 4–20% linear gradient SDS-PAGE mini-gels using a buffer system containing 25 mM Tris, 0.192 M glycine, and 0.1% SDS (Invitrogen, Grand Island, NY) and transferred to nitrocellulose in transfer buffer with 20% methanol at 4–15°C. For each sample, 1/10^th^ of a lens was loaded. For β2-spectrin and ankyrin-B, the gels were cut near the 100 kD marker, and the top portions were transferred without methanol in 0.01% SDS for 3 h at 4°C. The bottom portions were transferred in methanol and blotted for CP49, filensin, Tmod1, actin, γTM, and GAPDH. Transferred proteins were bound to the nitrocellulose by incubating in PBS for 1 h at 65°C. Blots were blocked in 4% BSA in PBS overnight at 4°C and incubated in primary antibodies overnight at 4°C. Rabbit polyclonal antibodies were as follows: antiserum to a unique C-terminal Tmod1 peptide, (residues 340–359, RKRRLADLTGPIIPKCRSGV-COOH; PA2211, 1∶5000; Open Biosystems, Lafayette, CO), antisera to CP49 (rabbit 899, 1∶2000) and filensin (rabbit 76, 1∶2000), and affinity-purified anti-ankyrin-B (4.4 µg/ml). Mouse monoclonal antibodies were as follows: anti-actin (C4, 10 ng/ml), anti-GAPDH (1∶5000; Novus Biologicals, Littleton, CO), anti-TM exon 9a (γTM, see [35]; TMCH1, 1.5 µg/ml; Developmental Studies Hybridoma Bank, Iowa City, IA), anti-β2-spectrin (42/B, 50 ng/ml; BD Biosciences, San Jose, CA), and anti-N-cadherin (3B9, 1∶1000; Invitrogen). After five 2-min washes with PBS +0.1% Triton X-100, blots were incubated for 1 h at room temperature in the following secondary antibodies: 800CW-conjugated goat anti-rabbit IgG (1∶20,000; LI-COR, Lincoln, NE) and 680LT-conjugated goat anti-mouse IgG (1∶20,000; LI-COR). After washing, bands were visualized using a LI-COR Odyssey infrared imaging system and quantified using ImageJ (http://rsbweb.nih.gov/ij) with normalization to GAPDH.

### Lens Immunostaining, Confocal Imaging, and Quantitative Image Analysis

Eyes were dissected from 1-mo-old mice, cut at the posterior pole to allow entry of fixative, and fixed in either 1% paraformaldehyde in PBS overnight at 4°C or in 0.75% paraformaldehyde in PBS for 4 h at room temperature for antibody and F-actin staining [35], [36]. Eyes were washed in PBS, cryoprotected in sucrose, frozen in OCT medium (Sakura Finetek, Torrance, CA) and stored at −80°C until use. Serial sections (12–14 µm thick) were cut in an equatorial orientation with a cryostat (Leica CM1950) and melted on ColorFrost Plus glass slides (Fisher Scientific, Pittsburgh, PA). Sections were then blocked and stained as previously described [35], [36] using mouse monoclonal anti-β2-spectrin (42/B, 0.5 µg/ml; BD Transduction Laboratories). The secondary antibody was Alexa-647-conjugated goat-anti-mouse IgG (1∶200; Invitrogen) supplemented with rhodamine-phalloidin (1∶200; Invitrogen) to stain F-actin and Hoechst 33258 (1∶500; Sigma-Aldrich, St. Louis, MO) to stain nuclei. After labeling and washing, slides were mounted using Gel/Mount (Biomeda, Foster City, CA). Images were acquired using a Bio-Rad Radiance 2100 laser scanning confocal microscope mounted on a Nikon TE2000-U microscope. Sections corresponding to the equatorial region of the lens were identified based on the thickness of the lens epithelium, which is maximal at the lens equator [35]. For fiber cell disorder measurements in the lens cortex, single optical sections of lens cryosections were acquired at room temperature using a 20×/0.75 NA-air objective lens (zoom 1) to examine fiber cell organization revealed by F-actin staining. Disordered fiber cell patches were manually outlined, and areas were measured using ImageJ. Area measurements were exported to Microsoft Excel to calculate percentages of the region of interest. For examination of spectrin-actin network integrity, a 100×/1.4 NA-oil objective lens (zoom 3) was used to examine β2-spectrin and F-actin distributions on fiber cell membranes. Z-stacks were collected using the 100× objective, with a step size of 0.3 µm, and ten 0.8-µm-thick optical sections were collected. Bio-Rad LaserSharp 2000 software was used during image collection. Three-dimensional image reconstructions were created using Volocity 5.3.2 (Improvision, Waltham, MA), and images were processed using Adobe Photoshop. All figures were constructed using Adobe Illustrator.

### Slit-lamp Ophthalmoscopy

Lenses from eyes of 14–16-mo-old, age-matched mice were dissected in DPBS at 37°C and photographed using oblique illumination on an Olympus SZ11 dissecting microscope with a Nikon CoolPix 990 camera. Aged animals were used in lens transparency experiments, as the opacification of CP49-null lenses becomes more pronounced with age [17], and pilot experiments found normal lens transparency in 2-mo-old mice of all genotypes (data not shown). Lens equatorial diameters were measured by including a ruler in the images. Lens transparency was examined *in situ* in eyes of non-anesthetized mice using a Nikon FS-2 slit-lamp ophthalmoscope, as previously described [18], [45]. Mouse eyes were dilated with a 1∶1 mixture of 1% tropicamide (Alcon, Fort Worth, TX) and 10% phenylephrine hydrochloride (Akorn, Abita Springs, LA). The slit-lamp angle was ∼40°, and the slit width was ∼0.2 mm. Examinations were recorded using a Canon Optura Pi digital camcorder, and slit-lamp images were captured using Adobe Premier and processed using Adobe Photoshop. Images were converted to grayscale in ImageJ, and a densitometric trace of pixel intensity was obtained across the anterior-posterior diameter of each lens. Pixel intensities were normalized to the maximum intensity of the cornea (set at 100%) and the minimum intensity of the aqueous chamber (set at 0%). The sum of normalized pixel intensities, mean normalized pixel intensity, and variance of normalized pixel intensity were determined from each normalized trace.

### Biomechanical Compression Testing Using Glass Coverslips

Unconfined compressive mechanical properties of 2-mo-old mouse lenses were assessed by resting successive glass coverslips onto immobilized lenses and monitoring lens shape changes under a dissecting microscope, using an approach adapted from methods described previously [46]. First, each lens was carefully dissected from its mouse eye and placed into a 200-µm-deep, circular, flat-bottomed divot located 1 cm from the edge of a custom-made acrylic testing chamber filled with DPBS (Fig. S1 A,B). Before loading, axial and sagittal images of the lens were collected using a digital camera mounted on a dissecting microscope to qualitatively assess tissue integrity and optical clarity. When acquiring axial images, the lens was located directly underneath the microscope objective. In contrast, when acquiring sagittal images, a reflection of the lens was viewed through a 45° mirror (face  = 5 mm × 5 mm) (Edmund Optics, Barrington, NJ) (Fig. S1A,B). The mirror was held at a constant distance from the divot to eliminate confounding linear perspective effects. An in-focus image of the mirror edge (length  = 5 mm) was also collected for distance calibration.

During compressive loading, 10 glass coverslips (18 mm × 18 mm; 129.3 mg each) were lined up along the edge of the testing chamber and carefully lowered onto the lens, one at a time, such that compression was predominantly applied along the anterior-posterior axis of the lens (Fig. S1C). (Note that, due to the oblique angle of the coverslips with respect to the floor of the chamber (θ), a small component of the applied load (sin θ**)** was applied equatorially instead of axially. However, we considered this effect to be negligible as the angle was ∼6° (sin θ ≈ 0.1).) After application of each coverslip, the lens was permitted to stress-relax and equilibrate for 2 min. A sagittal photograph was then acquired, and the next coverslip was applied. This procedure was repeated until all 10 coverslips were applied. Next, the lens was unloaded by carefully removing all 10 coverslips simultaneously, and axial and sagittal photographs were retaken to assess compression-induced tissue damage. Finally, the lens was decapsulated, cortical fiber cells were manually sloughed off by gently rolling the lens between the operator’s fingertips, and a sagittal image of the lens nucleus was collected.

During offline image analysis, axial and equatorial diameters of lenses and nuclei were measured in ImageJ (http://rsbweb.nih.gov/ij/). Axial diameters were corrected for the depth of the divot, which obscured 200 µm of the lens thickness. For normalization, axial and equatorial diameters were converted into strains using the equation ε = (*d*-*d_0_*)/*d_0_*, where ε is strain, *d* is the axial or equatorial diameter at a given load, and *d_0_* is the corresponding axial or equatorial diameter at zero load. Axial and equatorial strains were then plotted as functions of the imposed load (in mg). In addition, lens aspect ratio and volume were calculated as *r_eq_*/*r_ax_*, and (4/3)π*r_eq_* ^2^ *r_ax_*, respectively, where *r_eq_* and *r_ax_* are the corresponding equatorial and axial radii at zero load. Finally, the ratio of the nuclear diameter to the axial diameter was computed to determine the proportion of the axial diameter occupied by the nucleus. Note that the lens nucleus is effectively a rigid body when compared to the peripheral cortex; at the gram-level loading magnitude used in this study, the lens is incapable of undergoing additional axial compression once the nucleus occupies 100% of the lens’s axial diameter.

### Biomechanical Compression Testing Using a Dynastat Apparatus

The mechanical stiffness of 2-mo-old mouse lenses was also assessed using an unconfined compression procedure performed using a Dynastat mechanical loading apparatus (Dynastatics, Albany, NY) and high-resolution data acquisition methods adapted from those previously applied to bovine articular cartilage explants [47], [48]. Lenses were first dissected from mouse eyes in warmed DPBS, placed into storage medium (M199 supplemented with 5.56 mM glucose, 25 mM HEPES, 100 U/mL penicillin, and 100 mg/mL streptomycin), and stored in a 37°C CO_2_ incubator until use. Axial lens thickness was measured by resting the lens on top of a metal spacer adjacent to a right angle prism (6.4 mm × 6.4 mm; Rolyn Optics Company, Covina, CA). The entire setup was placed in a Petri dish full of medium at 37°C and photographed through an inverted microscope using a 2× objective. Lens thickness (in pixels) was converted into mm using a predetermined conversion factor for the objective (1 mm  = 272 pixels).

In pilot experiments, we determined that a 50 g load cell was sufficiently sensitive to detect stresses imposed by 5–20% axial compression regimes in lenses from 2-mo-old mice. The load cell had a resolution of 0.003 g when attached to the 16-bit data acquisition board used during mechanical testing; this load was found to be stable and sensitive for the measurements in the 0–0.8 g range. Prior to testing, the load cell was warmed up for at least 3 h, and a mechanical spectrometer and computer-controlled data acquisition system were warmed up for at least 1 hour. The lens was placed between a flat-ended, fluid-impermeable, stainless steel compressing piston that fully covered the sample and the base of the chamber. Prior to each test, 37°C storage medium was placed into the sample dish. The compressing piston imposed incremental compressions of 0%, 5%, 10%, 15%, 20%, 15%, 10%, 5%, and 0% of the lens thickness, with each ramp change in compression followed by 5 min of stress relaxation (Fig. S2). Force and deformation data were collected each second during each stress-relaxation phase and corrected for minor load cell drift during the course of the measurements (Fig. S2). Because the resulting load-compression curve was exponential in shape, exponential regression was applied. The lens’s compressive tangent modulus (an index of intrinsic compressive stiffness) was calculated as the derivative of the exponential load-compression curve at each level of compression. Hysteresis was assessed by comparing the load-compression curves during loading vs. unloading.

### Blot Overlay

Lens extracts were prepared, electrophoresed on 4–20% gradient mini-gels, and transferred to nitrocellulose, as described above. Transferred proteins were bound to the nitrocellulose by incubating the nitrocellulose in PBS for 1 h at 65°C, followed by two 2-min washes at 4°C in OverWash buffer (80 mM KCl, 2 mM MgCl_2_, 1 mM EGTA, 0.2% Triton X-100, 20 mM Hepes, pH 7.3). Blots were then incubated overnight at 4°C with 2 µg/ml of purified recombinant human Tmod1 [49] or chicken Tmod4 [50] in OverWash +2% BSA, and excess protein was rinsed with five 2-min washes with OverWash. (Negative control blots were incubated in OverWash +2% BSA alone.) Bound Tmods were then detected by incubating blots for 4 h at room temperature with, where appropriate, rabbit polyclonal antiserum to Tmod1 (PA2211, 1∶2500) or rabbit polyclonal antiserum to Tmod4 preadsorbed by passage through a human Tmod1 column (R3577, 1∶500). After five 2-min washes with OverWash, blots were incubated with 800CW-conjugated goat anti-rabbit IgG (1∶20,000) for 1 h at room temperature, washed again, and visualized with a LI-COR Odyssey imaging system, as described above.

### Coimmunoprecipitation (co-IP)

Six lenses from three 1- to 2-mo-old wild-type mice were dissected in DPBS, decapsulated, and placed into an ice-cold Dounce homogenizer containing 300 µl of lens homogenization buffer (100 mM NaCl, 1 mM MgCl_2_, 10 mM NaF, 2 mM EGTA, 20 mM Tris, 1 mM DTT, pH 7.4) supplemented with protease inhibitor cocktail (1∶1000, Sigma). After addition of 1.2 ml of modified RIPA buffer (150 mM NaCl, 1% NP-40, 0.5% sodium deoxycholate, 2 mM EGTA, 50 mM Tris-HCl, pH 7.4), lenses were homogenized and then centrifuged at 50,000 *g* for 30 min at 4°C. The resulting supernatant was divided into 650-µl aliquots, to which one of the following was added: 2 µg affinity-purified rabbit polyclonal anti-Tmod1 (R1749bl3c), 2 µl rabbit polyclonal antiserum to filensin (rabbit 76), or 2 µg preimmune control IgG. Antibody-bound protein complexes were adsorbed onto 100 µl of µMACS Protein A–conjugated super-paramagnetic MicroBeads (Miltenyi Biotec, Bergisch Gladbach, Germany) and incubated for 2 h at 4°C, followed by passage through a prewashed µColumn (Miltenyi Biotec) using a µMACS magnetic separator (Miltenyi Biotec). Beads were then washed 4× with modified RIPA buffer +1 mg/ml BSA, 4× with modified RIPA buffer alone, and 4× with PBS. Bound protein complexes were then eluted from the µColumn with 50 µl of hot 2× SDS sample buffer. The input extract (diluted in 300 µl of 5× SDS sample buffer) and eluted protein complexes were then subjected to SDS-PAGE and western blotting, as described above.

### Statistics

Data are presented as mean±SEM, and statistical significance was defined as *p*<0.05. Differences between two groups (with a single independent variable) were detected using Student’s *t*-test. Differences between three groups (with a single independent variable) were detected using one-way ANOVA with *post hoc* Fisher’s PLSD tests. Differences between four groups (with two independent variables) were detected using two-way ANOVA with *post hoc* Fisher’s PLSD tests. Statistical analysis was performed in Microsoft Excel and StatPlus:mac.

## Results

### Absence of CP49 Leads to Increased Actin, While Absence of Tmod1 Leads to Decreased Actin and γTM

To study the physiological significance of the spectrin-actin membrane skeleton in the lens, we examined the lens phenotype of the *Tmod1−/−^Tg+^* mouse [35], [40], [42], [43]. The *Tmod1−/−^Tg+^* mouse is on a mixed FvBN/129FvJ/C57Bl6 background with an endogenous mutation in the *Bfsp2/CP49* gene, resulting in the absence of CP49 (*CP49−/−*), concomitantly reduced levels of filensin, and no beaded filament cytoskeleton [18], [20], [35], [41]. Simultaneous absence of both Tmod1 and beaded filaments may have confounded the previously reported Tmod1-null lens phenotype and obscured the actual function of the spectrin-actin membrane skeleton in the lens. Therefore, to investigate the Tmod1-null lens phenotype in the presence of functional beaded filaments, we backcrossed *Tmod1−/−^Tg+^* mice with C57Bl6 mice to restore the wild-type *Bfsp2/CP49* allele (*CP49+/+*). Successful restoration of the *CP49+/+* allele was confirmed by PCR genotyping (Fig. 1A), and restoration of the CP49 protein in the resultant CP49-containing lenses was confirmed by western blotting of lens extracts (Fig. 1C). Consistent with previous work [18], [20], [35], [41], lenses from CP49-null mice completely lacked CP49 and had ∼1/5^th^ the normal level of filensin, with lenses lacking both CP49 and Tmod1 exhibiting identical decreases in filensin levels as lenses lacking CP49 alone (Fig. 1C–E). Importantly, CP49-null lenses had normal Tmod1 levels, and Tmod1-null lenses had normal CP49 levels (Fig. 1C,D,F), indicating that CP49 and Tmod1 do not depend on one another for achieving wild-type expression levels. Visual inspection of 2-mo-old dissected lenses revealed no cataracts or gross anatomical abnormalities arising from the absence of Tmod1 and/or CP49 (Fig. 1B), and no significant changes in lens axial and equatorial diameters, aspect ratios, or volumes were observed (Fig. S3 A–D).

Previously, we showed that deletion of Tmod1 from CP49-null lenses led to a ∼75% decrease in levels of γTM, but without effects on global levels of actin or other proteins in the spectrin-actin membrane skeleton [35]. To investigate whether loss of Tmod1 has a similar effect on the membrane skeleton in lenses containing CP49, we performed western blotting for actin, γTM, β2-spectrin, and ankyrin-B using Tmod1-containing and Tmod1-null lenses on both CP49-containing and CP49-null backgrounds. First, we observed that absence of Tmod1 led to a ∼30% reduction in actin levels in CP49-containing lenses, but this change was not observed in CP49-null lenses (Fig. 1C,G), in agreement with previous observations [35]. However, since deletion of CP49 led to a modest compensatory increase in actin, this may have obscured any reduction in actin due to loss of Tmod1 (Fig. 1C,G). Second, absence of Tmod1 led to a ∼30% decrease in γTM levels in the presence of CP49, and, in contrast to actin, the decrease in γTM levels was actually exacerbated in the absence of CP49 (Fig. 1C,H). Note that loss of CP49 alone had no effect on levels of γTM (Fig. 1C,H), and loss of CP49 and/or Tmod1 had no effect on the levels of β2-spectrin and ankyrin-B (Fig. 1C,I,J), consistent with previous observations [35]. Thus, absence of Tmod1 in a wild-type CP49 background leads to decreases in both actin and γTM levels, consistent with a function for Tmod1 in stabilizing the actin cytoskeleton. These biochemical consequences of Tmod1 deletion appear to have a subtle dependence on beaded filaments, with the decrease in actin blunted and the decrease in γTM exacerbated in a CP49-null background. Conversely, while loss of CP49 results in an increase in overall actin levels, loss of CP49 has no effect on levels of γTM, β2-spectrin, and ankyrin-B.

**Figure 2:**
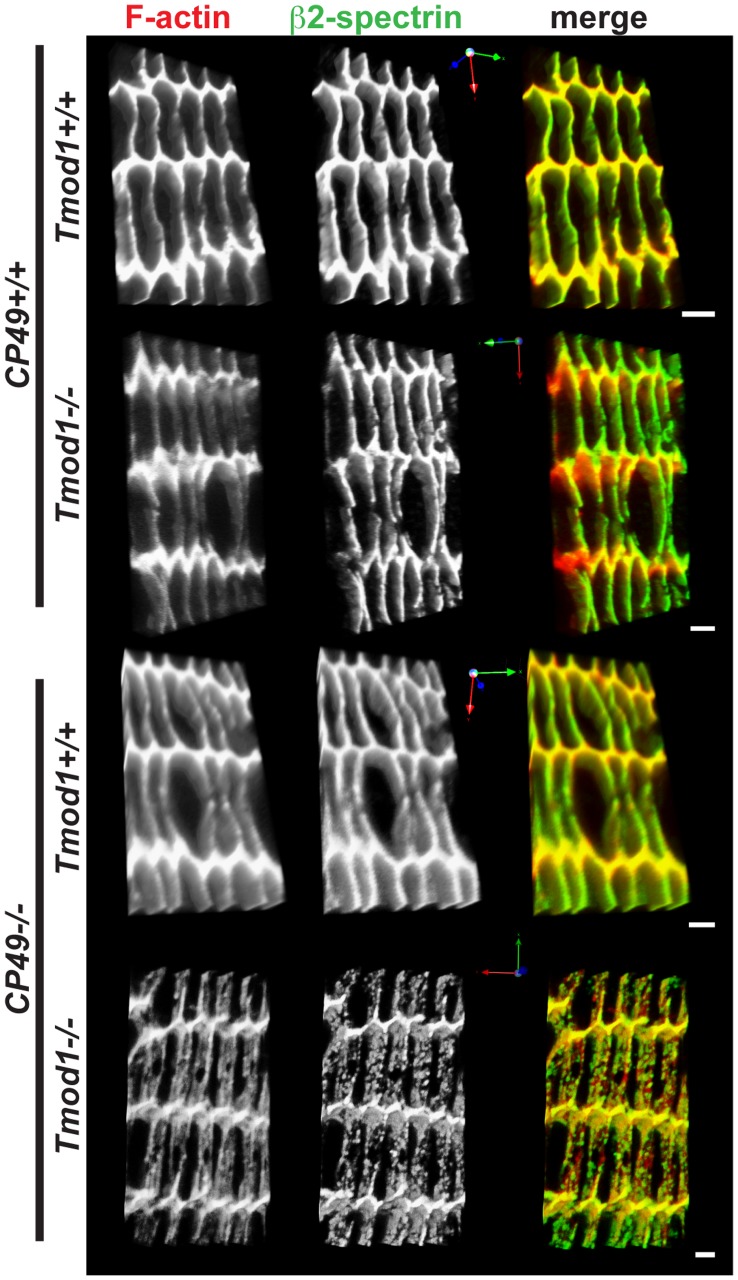
Simultaneous deletion of both Tmod1 and CP49 leads to widespread disruption of F-actin and β2-spectrin organization on lens fiber cell membranes. Panels depict three-dimensional reconstructions of confocal Z-stacks of equatorial cryosections from 1-mo-old *Tmod1+/+* and *Tmod1−/−* mouse lenses in both *CP49+/+* and *CP49−/−* backgrounds. Cryosections were immunostained for β2-spectrin and phalloidin-stained for F-actin. Spectrin-actin network organization was examined in equatorial sections from 3–4 animals from each genotype, and representative images are shown. Scale bars, 2 µm.

To investigate whether loss of Tmod1 and/or CP49, with concomitant alterations in cytoskeletal protein levels, leads to altered spectrin-actin network integrity, we used confocal microscopy to examine F-actin and β2-spectrin localization on fiber cell membranes in equatorial sections of lenses lacking Tmod1 and/or CP49. These experiments showed a predominantly smooth and continuous (or only very slightly disrupted) spectrin-actin network associated with fiber cell membranes in wild-type, Tmod1-null, and CP49-null lenses (Fig. 2). By contrast, simultaneous deletion of Tmod1 and CP49 did lead to widespread disruptions in the spectrin-actin network (Fig. 2), consistent with previous findings [35]. Notably, in lenses lacking only Tmod1, we observed redistribution of F-actin away from the broad fiber cell sides and toward the short sides and vertices (Fig. 2), which occurred in parallel with reduced total actin in these lenses (Fig. 1C,G). Thus, Tmod1 and beaded filaments synergistically maintain spectrin-actin network integrity in the mouse lens.

### Tmod1 and CP49 Synergistically Regulate Lens Fiber Cell Organization and Light Scattering

**Figure 3:**
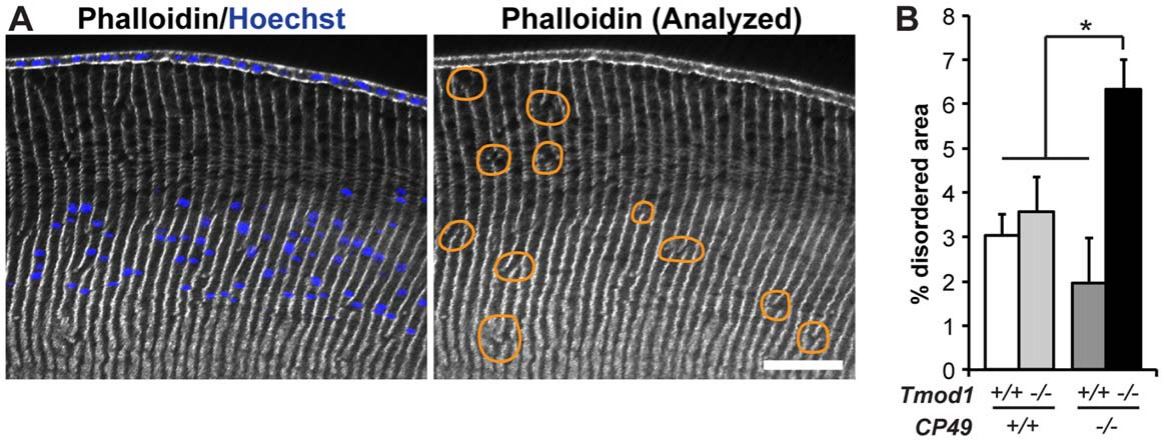
Simultaneous deletion of both Tmod1 and CP49 increases the sizes of disordered fiber cell areas in the lens cortex. (A) Sample low-magnification images of equatorial cryosections of a 1-mo-old mouse lens. Cryosections were phalloidin-stained for F-actin to reveal columnar fiber cell organization and Hoechst-stained for nuclei to indicate cortical localization. Areas with disrupted radial columns, indicated by discontinuities in vertical F-actin stripes, are outlined with orange lines. Scale bar, 58 µm. (B) Percentage of disordered fiber cell area in the cortex of *Tmod1+/+* and *Tmod1−/−* mouse lenses in both *CP49+/+* and *CP49−/−* backgrounds. The sum of individual areas was normalized to the total analyzed area for each image and region and expressed as a percentage. Error bars reflect mean±SEM of *n* = 16−18 images/genotype collected from 6 lenses/genotype. *, *p*<0.05.

Previous studies have shown that the spectrin-actin membrane skeleton and beaded filament cytoskeleton are critical regulators of membrane stability and hexagonal fiber cell packing in the mouse lens [19], [21], [35], [36]. To examine whether beaded filaments synergize with the spectrin-actin network in regulating fiber cell geometry in the mouse lens, we performed quantitative analysis of cortical fiber cell disorder in lenses lacking Tmod1 and/or CP49 (Fig. 3A). We performed our analysis on cortical fiber cells located up to 250 µm in from the epithelium, up to and encompassing the region where nuclei break down [36]. (Note that this approach does not address the organization of the older fiber cells deeper in the lens.) While our analysis showed that deletion of Tmod1 or CP49 alone does not adversely impact the low level of fiber cell disorder in the lens cortex, a higher degree of fiber cell disorder was evident in lenses lacking both Tmod1 and CP49 (Fig. 3B). Thus, Tmod1 and CP49 synergistically regulate cortical fiber cell organization in the mouse lens.

**Figure 4:**
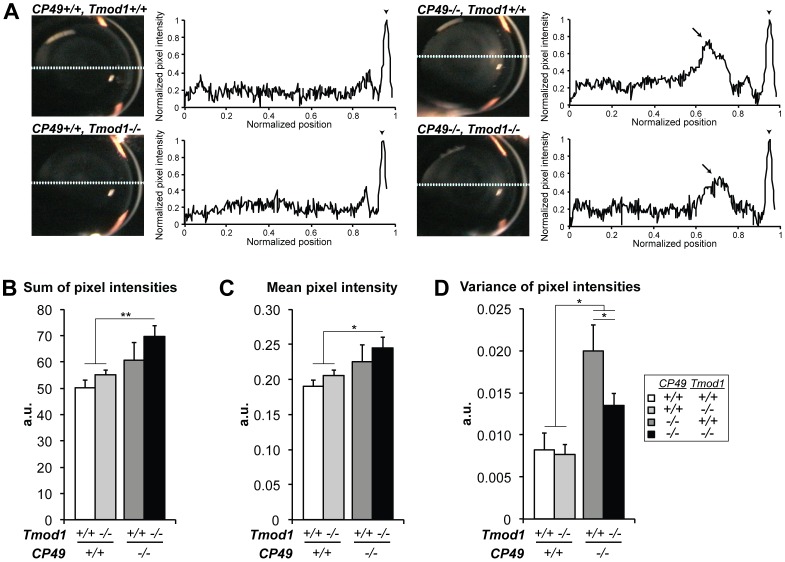
Tmod1 and CP49 synergistically regulate light scattering through the lens, as determined by slit-lamp ophthalmoscopy. (A) Sample *in situ* images and densitometric analysis of line scans obtained from slit-lamp ophthalmoscopy of 14–16-mo-old mouse eyes, revealing peaks of opacification (arrows) in the outer nuclear region in *CP49−/−* lenses. Pixel intensities were normalized to the maximum intensity of the cornea (arrowheads) and the minimum intensity of the aqueous chamber. (B) The sum of pixel intensities and (C) the mean pixel intensity were both significantly increased in lenses lacking both Tmod1 and CP49, but not in lenses lacking Tmod1 or CP49 alone. (D) Loss of CP49 significantly increased the variance of pixel intensity in *Tmod1+/+* lenses, but this was partially rescued by simultaneous deletion of Tmod1. Error bars reflect mean±SEM of *n* = 8−10 lenses/genotype. *, *p*<0.05; **, *p*<0.01.

To test whether defects in fiber cell organization impact the optical properties of lenses lacking CP49 and Tmod1, we subjected 14–16-mo-old mouse lenses lacking Tmod1 and/or CP49 to slit-lamp ophthalmoscopy and measured light scattering across line scans of video stills [45]. Line scans of CP49-null lenses revealed peaks of increased light scattering, indicative of opacification that was not present in wild-type lenses, as expected [17], [18], [19], but, intriguingly, simultaneous deletion of Tmod1 reduced the magnitude of these peaks (Fig. 4A). Quantification of the line scans showed that the sum of pixel intensities and the mean pixel intensity (two indices of total light scattering) both trended towards being increased in lenses lacking either CP49 or Tmod1, but these trends failed to achieve statistical significance (Fig. 4B,C). It is noteworthy that simultaneous deletion of both CP49 and Tmod1 did significantly increase total light scattering compared to wild-type lenses (Fig. 4B,C), consistent with the increased fiber cell disorder in CP49-null/Tmod1-null lenses (Fig. 3B). Furthermore, consistent with the presence and magnitude of the enlarged light scattering peaks in CP49-null lenses, deletion of CP49 alone did significantly increase the variance of pixel intensity across the line scans (an index of heterogeneity of light scattering). Interestingly, the increased variance of pixel intensity in CP49-null lenses was partially rescued by simultaneous deletion of Tmod1 (Fig. 4D), although the mechanism for this rescue is uncertain (see Discussion).

### Tmod1 and CP49 both Regulate Lens Stiffness, but at Distinct Magnitudes of Applied Load

Disruption of the spectrin-actin membrane skeleton (e.g., via Tmod1 deletion) is known to mechanically weaken the RBC membrane by interfering with membrane stiffness and extensibility [37], [38], [40]. Likewise, perturbing the intermediate filament cytoskeleton (e.g., via CP49 deletion) mechanically weakens the lens by eliminating load-bearing by functional beaded filaments [22]. To analyze the simultaneous biomechanical roles of the spectrin-actin membrane skeleton and beaded filament cytoskeleton in the intact lens and probe for potential functional synergy between these networks, we modified an established glass coverslip-based axial compression technique [46] and measured changes in the biomechanical compressive properties of 2-mo-old lenses lacking Tmod1 and/or CP49. To account for any variations in lens size during the compression experiments, all changes in the equatorial and axial dimensions of loaded lenses were normalized to the lenses’ unloaded dimensions and expressed as equatorial and axial strains, respectively. Moreover, deletion of Tmod1 and/or CP49 did not affect the diameter of the rigid lens nucleus, or the proportion of the axial diameter occupied by the nucleus (nuclear/axial diameter), indicating that Tmod1 and CP49 levels do not impact the relative mechanical contributions of the deformable lens cortex and rigid lens nucleus during compression testing (Fig. S3 E,F).

**Figure 5:**
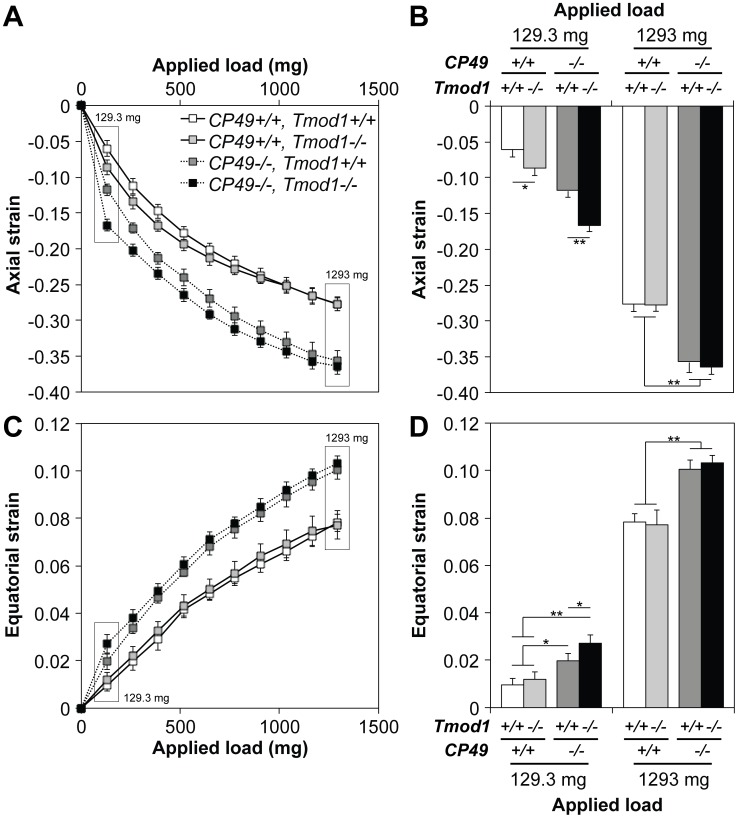
Tmod1 and CP49 synergistically regulate the ability of the lens to bear compressive load, as determined by a coverslip-based compression assay. (A) Axial strain-load curves of 2-mo-old mouse lenses lacking Tmod1 and/or CP49 reveal logarithmic strain-load relationships. (B) At a low load of 129.3 mg (1 coverslip), *Tmod1−/−* lenses exhibited more axial strain than *Tmod1+/+* lenses, on both *CP49+/+* and *CP49−/−* backgrounds. In contrast, at a high load of 1293 mg (10 coverslips), *CP49−/−* lenses exhibited more axial strain than *CP49+/+* lenses, regardless of the presence of Tmod1. (C) Equatorial strain-load curves of 2-mo-old mouse lenses lacking Tmod1 and/or CP49 reveal logarithmic strain-load relationships. Equatorial extension was a Poisson effect of axial compression. (D) At a low load of 129.3 mg (1 coverslip), *Tmod1−/−* lenses on a *CP49−/−* background exhibited more equatorial strain than *Tmod1+/+* lenses. At a high load of 1293 mg (10 coverslips), *CP49−/−* lenses exhibited more equatorial strain than *CP49+/+* lenses, regardless of the presence of Tmod1 (identical to axial strain). Error bars reflect mean±SEM of *n* = 8 lenses/genotype. *, *p*<0.05; **, *p*<0.01.

In the coverslip-based compression tests, axial strain was a logarithmic function of the applied load, regardless of the presence or absence of CP49 or Tmod1 (Fig. 5A). At a low load of 1 coverslip (129.3 mg) and in the presence of Tmod1, CP49-null lenses exhibited higher strain (less stiffness) than CP49-containing lenses, consistent with previous data on lenses with no beaded filaments [22] (Fig. 5B). Likewise, deletion of Tmod1 caused a marked increase in axial strain in the presence of CP49, but the magnitude of this increase was more pronounced in the absence of CP49 (Fig. 5B). Thus, at low loads, both the spectrin-actin membrane skeleton and beaded filament cytoskeleton contribute to lens stiffness, but the relative importance of the membrane skeleton is heightened when the beaded filament cytoskeleton is compromised. By contrast, at a high load of 10 coverslips (1293 mg), CP49-null lenses continue to show higher strain than CP49-containing lenses, in agreement with previous data [22], but the effect of Tmod1 deletion on lens stiffness is eliminated, regardless of the presence of CP49 (Fig. 5B). Our maximum load of 10 coverslips was indeed sufficient to induce deformation of the entire lens cortex, as indicated by the fact that ∼100% of the axial diameter was occupied by the nuclear diameter upon application of 10 coverslips (up from ∼65% in unloaded lenses) (Fig. S3 F). Together, these data suggest that the spectrin-actin membrane skeleton does not have a meaningful influence on lens stiffness at high loads, while the beaded filament cytoskeleton does.

To lend further credence to this result, we assessed the equatorial extension that occurs during axial compression as a Poisson effect to conserve lens volume. The effects of Tmod1 and CP49 levels on equatorial strain were nearly identical to those observed for axial strain, with the minor exception of Tmod1 deletion having no effect on equatorial strain at a low load of 1 coverslip (129.3 mg) in the presence of CP49 (Fig. 5C,D). However, it should be noted that the maximum measured equatorial strain magnitude (∼0.1) was considerably less than the maximum measured axial strain magnitude (∼0.35), indicating less resolution to measure minute incremental strains in the equatorial direction. Moreover, to determine whether the coverslip-based loading procedure induced permanent plastic deformation in the lens, we re-measured lens geometry after unloading. We observed no changes in lens diameters, aspect ratios, and volumes after the maximum load of 10 coverslips was removed, indicating the absence of permanent, compression-induced plastic deformation (Fig. S3 A–D).

**Figure 6:**
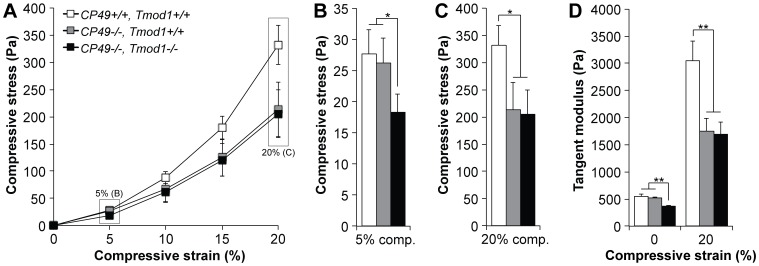
Tmod1 and CP49 synergistically regulate lens stiffness, as determined by a high-resolution Dynastat-based compression protocol. (A) Compressive stress-strain curves of 2-mo-old wild-type, *CP49−/−*, and *CP49−/−;Tmod1−/−* mouse lenses reveal exponential stress-strain relationships. (B) At a low strain of 5%, *CP49−/−;Tmod1−/−* lenses bore less stress than wild-type or *CP49−/−* lenses. (C) At a high strain of 20%, *CP49−/−* and *CP49−/−;Tmod1−/−* lenses bore less stress than wild-type lenses. (D) At zero strain, the instantaneous (tangent) modulus of *CP49−/−;Tmod1−/−* lenses was lower than that of wild-type or *CP49−/−* lenses. By contrast, at 20% strain, the tangent moduli of both *CP49−/−* and *CP49−/−;Tmod1−/−* lenses were lower than that of wild-type lenses. Error bars reflect mean±SEM of *n* = 8−10 lenses/genotype. *, *p*<0.05; **, *p*<0.01.

While the coverslip-based compression assay clearly demonstrated that CP49 regulates lens stiffness at both low and high loads, with Tmod1 only regulating stiffness at low loads, this assay was only designed for quantifying deformation as a function of applied load. To perform the inverse experiment (i.e., quantifying load as a function of applied deformation), we measured the stiffness of isolated 2-mo-old mouse lenses using an unconfined axial compression protocol in a Dynastat apparatus [47], [48]. In the Dynastat-based compression tests, compressive stress was an exponential function of the applied compressive strain for all lenses (Fig. 6A), consistent with the logarithmic strain-load curves obtained in the coverslip-based compression assays (Fig. 5A). Deletion of CP49 had no effect on load-bearing at low (5%) strain, but simultaneous deletion of both CP49 and Tmod1 resulted in lenses bearing ∼30% less stress at low strain (Fig. 6B). In contrast, deletion of CP49 resulted in a dramatic, ∼40% decrease in stress at high (20%) strain, which was not exacerbated by simultaneous deletion of Tmod1 (Fig. 6C). These observations were corroborated by computing the instantaneous (tangent) moduli of lenses at various strains. At zero strain, deletion of Tmod1 resulted in a ∼32% reduction in the tangent modulus in the absence of CP49 (Fig. 6D). However, at 20% strain, deletion of CP49 resulted in a ∼43% reduction in the tangent modulus, which was unaffected by simultaneous deletion of Tmod1 (Fig. 6D). No lenses of any genotype exhibited hysteresis, as demonstrated by identical loading and unloading curves (Fig. S4). Together, these mechanical data argue for a model in which the spectrin-actin membrane skeleton is a regulator of lens stiffness at low loads, while the beaded filament cytoskeleton primarily contributes to less stiffness at high loads. Note that the precise contribution of the intermediate filament cytoskeleton at low loads remains inconclusive, given the inconsistent effects of CP49 deletion in the low-load scenarios in the coverslip-based and Dynastat-based compression experiments (Fig. 5B, 6B).

### Interaction between Tmod1 and Beaded Filament Proteins

**Figure 7:**
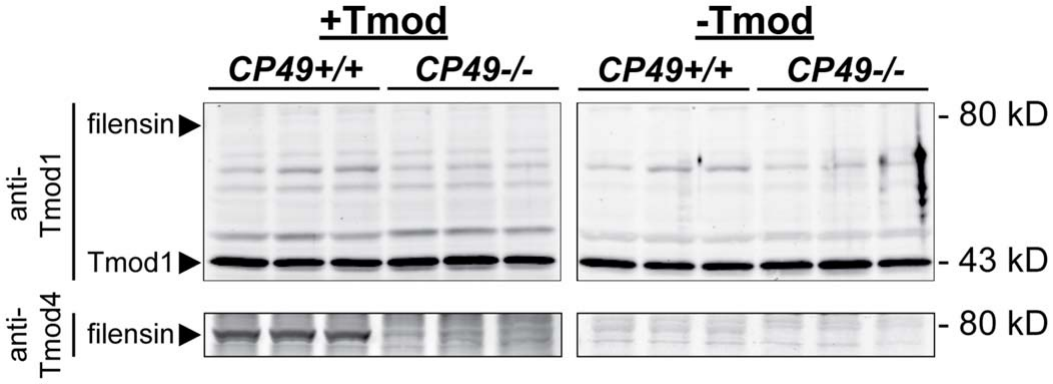
Tmod1 does not bind filensin in a blot overlay assay. *CP49+/+* and *CP49−/−* mouse lens extracts were separated by SDS-PAGE and transferred to nitrocellulose. Blots were pre-incubated in the presence (+Tmod) or absence (-Tmod) of 2 µg/ml Tmod1 (upper panels) or 2 µg/ml Tmod4 (lower panels). Tmod-labeled blots were then probed with antibodies recognizing either Tmod1 or Tmod4, as indicated, to reveal endogenous Tmod1 (∼43 kD) and recombinant Tmod4 (but not Tmod1) bound to mouse filensin at ∼80 kD (arrowheads). The Tmod4-binding band at ∼80 kD was dramatically reduced in *CP49−/−* extracts, confirming its identity as filensin, since *CP49−/−* lenses contain only 1/5^th^ of wild-type filensin levels (Fig. 1 and [18], [20], [41]). The ∼43 kD band in the upper panels corresponds to endogenous Tmod1 labeled by the anti-Tmod1 antibody.

In chicken lenses, Tmod4 binding to filensin provides a molecular linkage between the spectrin-actin membrane skeleton and beaded filament cytoskeleton [51]. However, mammalian lenses lack Tmod4 and contain Tmod1 in the membrane skeleton [28]. Hence, we speculated that Tmod1 might also bind filensin in mouse lenses in a manner analogous to Tmod4 binding filensin in chicken lenses, with Tmod1/filensin binding explaining the functional synergy between the membrane skeleton and beaded filament cytoskeleton in mouse lenses. To test this possibility, we performed blot overlay assays using mouse lens extracts preincubated with purified recombinant Tmods followed by the appropriate antibodies to detect the bound Tmods. In this experiment, the anti-Tmod1 antibody labeled endogenous Tmod1 at the expected molecular weight of 43 kD, as expected, regardless of whether the blots were preincubated with Tmod1 (Fig. 7). However, an antibody-labeled band corresponding to mouse filensin at the expected molecular weight of ∼80 kD was not detected in the samples from *CP49+/+* lenses that were pre-incubated with Tmod1 (Fig. 7). Binding of Tmod1 to CP49 was also not detected (Fig. 7). As a positive control, we confirmed that exogenous Tmod4 bound to an ∼80 kD protein band corresponding to mouse filensin (Fig. 7), whose intensity was dramatically reduced in *CP49−/−* lenses containing only 1/5^th^ of wild-type filensin levels (Fig. 1 and [18], [20], [41]). Tmod4 binding to mouse filensin agrees with previous findings showing Tmod4 binding to bovine and chicken filensin [51], but this interaction is physiologically irrelevant to the mouse lens, due to the lack of Tmod4 expression in mammalian lenses [28]. We conclude that direct binding of Tmod1 (in the spectrin-actin membrane skeleton) to filensin (in the beaded filament cytoskeleton) cannot account for the functional synergy between the spectrin-actin membrane skeleton and beaded filament cytoskeleton in the mouse lens.

**Figure 8:**
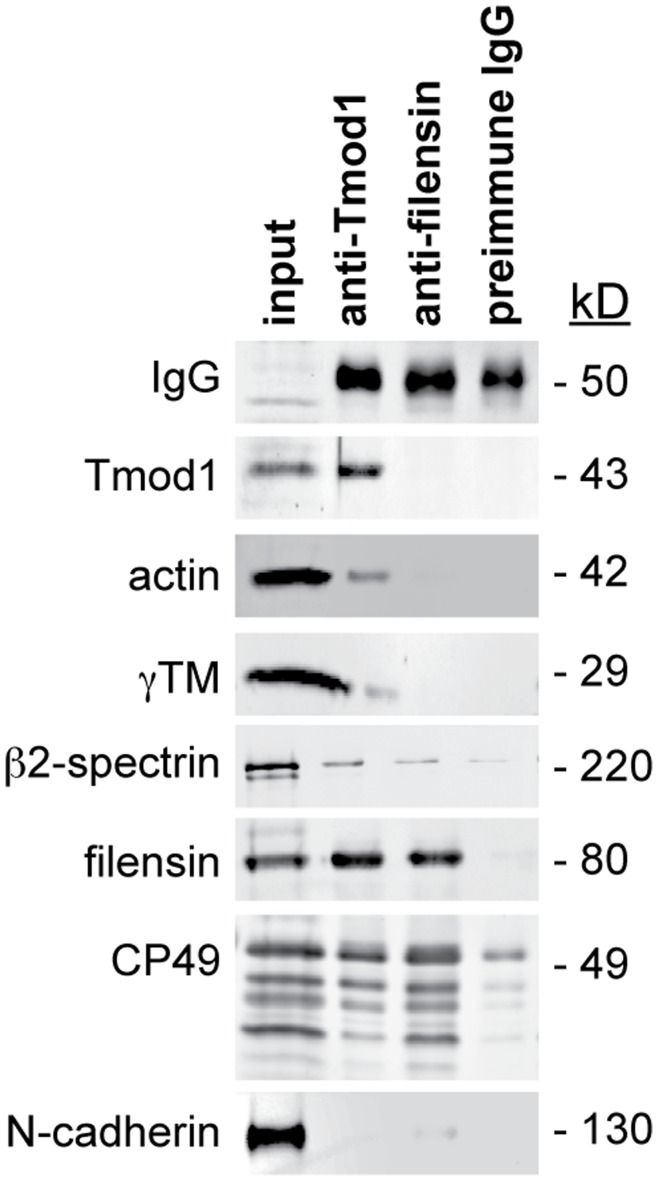
Beaded filament proteins are in a complex with Tmod1, as determined by a co-IP assay. Panels show western blots of mouse lens extracts (input) co-IP’ed using an antibody against Tmod1, an antiserum against filensin, or a preimmune (negative control) antibody. Note the co-IP of Tmod1, actin, γTM, β2-spectrin, CP49, and filensin, but not N-cadherin. As expected, IgG was present in all co-IP’s but not in the input extract.

The lack of direct binding between Tmod1 and filensin leaves open the possibility that an indirect biochemical association may account for the functional synergy between the spectrin-actin network and beaded filament cytoskeleton in the mouse lens. Therefore, to test whether Tmod1 could be indirectly associated with beaded filament proteins in the mouse lens, we performed co-IP experiments using wild-type mouse lens extracts. As expected, actin, γTM, and β2-spectrin co-IP’ed with anti-Tmod1-coated beads, but, importantly, CP49 and filensin were also observed to co-IP with anti-Tmod1-coated beads (Fig. 8). By contrast, N-cadherin failed to co-IP with anti-Tmod1-coated beads (Fig. 8). In an inverse experiment, only filensin, CP49, and β2-spectrin co-IP’ed with anti-filensin-coated beads, while actin, γTM, and Tmod1 did not co-IP with anti-filensin-coated beads (Fig. 8). The ability of anti-filensin-coated beads to co-IP filensin and CP49 but not actin, γTM, or Tmod1 was most likely due to a large excess of beaded filaments in the lens as compared to Tmod1-capped actin filaments (data not shown). Moreover, a control co-IP using preimmune IgG demonstrated the specificity of the associations of actin, γTM, β2-spectrin, CP49 and filensin with the anti-Tmod1 antibody (Fig. 8). Thus, an indirect biochemical association between Tmod1 and beaded filament proteins via an as-yet-unidentified adaptor protein may underlie the functional synergy between the spectrin-actin membrane skeleton and beaded filament cytoskeleton in the mouse lens.

## Discussion

In this study, we analyzed the phenotypes of *Tmod1+/+* and *Tmod1−/−* lenses from mice on both *CP49+/+* and *CP49−/−* backgrounds and discovered a novel physiological synergy between the spectrin-actin membrane skeleton and beaded filament cytoskeleton that is important for regulating lens fiber cell organization, transparency, and mechanical stiffness. How do the localizations and biochemistry of these networks relate to their overlapping physiological functions? The components of the spectrin-actin membrane skeleton localize to the membranes of hexagonally packed fiber cells during fiber cell maturation in the lens cortex, with F-actin enriched at the vertices and Tmod1, γTM, and β2-spectrin showing more uniform distributions along the short and long sides of the fiber cells [30], [35]. The beaded filament proteins CP49 and filensin also localize to fiber cell membranes, but mainly in the younger fiber cells in the shallow cortex, while beaded filament proteins in the older cells in the deep cortex exhibit proteolytic cleavage and more diffuse cytoplasmic localization [13], [14], [15]. Consistent with the membrane associations of the spectrin-actin membrane skeleton and beaded filament cytoskeleton in the lens cortex, our co-IP experiments reveal the association of Tmod1 in a complex with CP49 and filensin that also contains actin, γTM, and β2-spectrin. Thus, the spectrin-actin membrane skeleton and beaded filament cytoskeleton are biochemically linked (Fig. 8), despite the lack of direct binding between Tmod1 and the beaded filament proteins, filensin and CP49 (Fig. 7). It is likely that an as-yet-undiscovered molecular adaptor indirectly links the Tmod1-capped actin filaments of the spectrin-actin network to the beaded filament cytoskeleton. While both membrane skeleton and beaded filament proteins also co-IP as constituents of the ezrin/periplakin/periaxin/desmoyokin (EPPD) and cadherin-based complexes in the cortex adherens of the lens fiber cell membrane [52], [53], the proximities and interactions of the individual proteins within these large complexes are uncertain. Namely, co-IP approaches depend on the variable stringencies of the detergents used in the various IP buffers and may identify large complexes with many indirectly associated components, particularly in the case of the F-actin and intermediate filament polymers in the cytoskeleton. We speculate that crosslinker molecules capable of binding multiple cytoskeletal filament types, such as spectraplakins [54] and plectins [55], may directly tether F-actin to beaded filaments in the mouse lens, but identification of such crosslinkers is beyond the scope of this study.

### Tmod1 and CP49 Synergistically Regulate Lens Fiber Cell Organization and Transparency

Our data make the novel finding that Tmod1 and CP49 cooperate to establish and maintain fiber cell organization during fiber cell elongation and maturation throughout the lens cortex. While the absence of Tmod1 alone has no effect on fiber cell organization in the cortex of *CP49+/+* lenses (Fig. 3), a requirement for Tmod1 in promoting ordered fiber cell packing in the absence of CP49 was confirmed [35], [36]. Notably, the total disordered cortical area was ∼2–3% in wild-type, Tmod1-null, and CP49-null lenses, but the simultaneous absence of Tmod1 and CP49 results in an increase to ∼6% (Fig. 3), suggesting functional cooperativity between these two cytoskeletal systems in maintaining fiber cell organization in the lens cortex. Thus, the fiber cell interactions that maintain hexagonal packing geometry appear to be sufficiently robust to withstand the cytoskeletal disruptions due to loss of Tmod1 or CP49 alone.

While our data are generally consistent with extensive work highlighting the importance of beaded filaments in the maintenance of optical clarity and prevention of age-dependent cataract [16], [17], [18], [19], [20], some noteworthy discrepancies were revealed by our quantitative analysis of high-resolution slit-lamp ophthalmoscopy, which enabled us to study the optical properties of mutant mouse lenses *in situ* [45]. First, we found that aged CP49-null lenses exhibit peaks of opacification in the outer nuclear region, but these peaks only manifested as increased variance of light scattering along the lens diameter, with statistically insignificant trends toward increased mean or total light scattering (Fig. 4B,C). Second, simultaneous deletion of both CP49 and Tmod1 was required to induce significant increases in mean and total light scattering, but this also resulted in a partial rescue of the increased variance of light scattering exhibited by lenses lacking only CP49 (Fig. 4D). While the mechanism for this rescue is unclear, we suspect that spectrin-actin network disruption induced by Tmod1 deletion may permit molecular rearrangements that do not occur when the spectrin-actin network is intact, leading to enhanced turnover and clearance of cytoplasmic content harboring pathologically variable light scattering properties. Enhanced molecular rearrangements may also account for why Tmod1 deletion induces a more pronounced decline in γTM levels in CP49-null lenses than in CP49-containing lenses (Fig. 1C,H).

Our measurements of fiber cell organization and optical properties provide a means to answer the question of whether defects in hexagonal fiber cell packing can directly account for observed opacification, as predicted previously [1]. While increased cortical fiber cell disorder in lenses lacking both Tmod1 and CP49 [36] is indeed associated with increased mean and total light scattering (Fig. 4B,C), this increased disorder fails to correlate with the reduced variance of light scattering (Fig. 4D). By contrast, lenses lacking only CP49 demonstrate a marked increase in the variance of light scattering without a correspondingly robust increase in fiber cell disorder, while lenses lacking only Tmod1 demonstrate normal light scattering in the presence of unchanged levels of fiber cell disorder (Fig. 3,4). Thus, lens transparency tolerates small defects in cortical fiber cell organization, and loss of transparency is not necessarily due to increases in fiber cell disorder. Therefore, how does combined deletion of Tmod1 and CP49 induce defects in lens transparency, if not via regulation of fiber cell organization? While the exact mechanism(s) remain to be elucidated, we speculate that disruption of the spectrin-actin membrane skeleton or loss of the beaded filament cytoskeleton (occurring downstream of Tmod1 or CP49 deletion, respectively) creates aberrant “voids” in the submembrane region of the lens fiber cell cytoplasm, which may serve as nucleation hotspots for the precipitation of crystallin aggregates. Additional biochemical experiments assessing the effects of lens cytoskeletal proteins on crystallin solubility are required to address this hypothesis.

The synergistic effects of simultaneous Tmod1 and CP49 deletion on lens fiber cell organization and transparency are reminiscent of the abnormal fiber cell shapes, loss of hexagonal packing geometry and loss of transparency that are observed in lenses from mice with targeted disruptions in other structural and signaling pathways. These include the axon guidance molecules, ephrin-A5 ligand [56] and EPHA2 receptor [57], the cytoskeletal scaffold, periaxin [52], the spectrin-actin membrane adaptor, ankyrin-B [33], and one of ankyrin-B’s membrane attachment points, NrCAM [33]. However, in these systems, both the extent of fiber cell disorder and the loss of transparency have only been assessed qualitatively, and quantitative approaches such as those described here would be useful in comparing the lens phenotypes of these transgenic mice. It is also noteworthy that disruption of the membrane-associated F-actin via targeted Tmod isoform depletion is an effective strategy for perturbing membrane shape and stability in a variety of non-lens cell types, including polarized epithelial cells, RBCs, and skeletal muscle fibers [31], [40], [58], [59], [60]. These studies broadly point to a sophisticated array of molecularly distinct but functionally overlapping protein networks that cooperate to direct membrane morphology in both the lens and elsewhere, all of which converge on the Tmod-capped actin filaments of the spectrin-actin membrane skeleton.

### Tmod1 and CP49 Synergistically Regulate Lens Biomechanics

This study has revealed important roles for the spectrin-actin membrane skeleton and beaded filament cytoskeleton in regulating the mechanical stiffness of the mouse lens. In both our coverslip- and Dynastat-based compression experiments, deletion of Tmod1 resulted in reduced load borne at low axial strains, while deletion of CP49 resulted in reduced load borne at high axial strains, with concomitant reductions in the instantaneous stiffness (tangent modulus) at each of these strains (Fig. 5,6). These data point toward a model in which the spectrin-actin membrane skeleton mechanically fortifies the lens at low loads and strains, while beaded filaments provide fortification at high loads and strains. Therefore, it is likely that the distinct cytoskeletal architectures of the lens epithelium, superficial cortex, deep cortex, and nucleus give rise to radially variable and depth-dependent recruitment of cytoskeletal scaffolding during tissue compression, resulting in radial heterogeneity in the compressive modulus. Such a model is analogous to biomechanical characterization of articular cartilage, in which the depth-dependence of the compressive modulus and its correlation with aging and biochemical composition have been studied in detail [48], [61], [62]. Additional support for this model comes from our measurements of the whole-lens tangent moduli of wild-type lenses (∼500 Pa at zero compression and ∼3000 Pa at 20% compression), which fall between the individual moduli of isolated sheep lens cortical fiber cells (∼220 Pa) and nuclear fiber cells (∼4800 Pa) determined by atomic force microscopy [63]. Our tangent moduli are also comparable to the moduli of pig, cow, and monkey lenses determined using a variety of techniques, including whole-lens compression, microindentation, and atomic force microscopy [64], [65], [66]. Thus, the mammalian lens appears to exhibit a radial stiffness gradient, with cortical fiber cells gradually stiffening as they age and become displaced inwards toward the lens nucleus.

Our data also confirm and extend the results of a previous study by Fudge *et al.*, who found that deletion of CP49 leads to reduced lens stiffness [22]. However, Fudge *et al.* also observed slightly increased resilience in CP49-null lenses (calculated as the percent change in the area under the load-compression curve during loading vs. unloading), which is a biomechanical phenotype that we did not observe when comparing the loading and unloading curves (hysteresis loops) of wild-type and CP49-null lenses (Fig. S4). The most likely explanation for this difference is that Fudge *et al.* used a dynamic “sawtooth” loading and unloading procedure, whereas our study used quasistatic procedures with stress-relaxation periods between incremental deformations. As a result, our study reports equilibrium loads while excluding confounding viscous effects, while the loads reported by Fudge *et al.* incorporate a nontrivial viscous component. Viscous effects may also account for the linear load-compression relationships reported by Fudge *et al.*, which differ from the exponential relationships reported here. Indeed, the viscoelastic behaviors of intermediate filament networks are complex and specialized for their diverse mechanical milieus [67]. Hence, additional studies are required to fully characterize the depth-dependent viscous and elastic stiffness coefficients of mammalian lenses and relate these mechanical parameters to lens fiber cell organization, transparency, and cytoskeletal architecture.

## Supporting Information

Figure S1 **Experimental setup for coverslip-based compression testing of mouse lenses.** (A) Schematic and (B) photograph of the experimental setup depict a mouse lens in a 200-µm-deep divot in an acrylic chamber filled with DPBS. To acquire sagittal images using a digital camera mounted on a dissecting microscope, a reflection of the lens was viewed through a 45° mirror. Compression was applied by lining up 10 coverslips along the edge of the chamber and carefully lowering them onto the lens, one at a time. Application of each coverslip was followed by 2 min of stress relaxation. (C) Photographs of sagittal views of lenses compressed by successively increasing numbers of coverslips provided the raw data for measuring axial and equatorial diameters and calculating axial and equatorial strains during coverslip-based compression testing. Axial diameters were corrected for the depth of the divot, which obscured 200 µm of the lens thickness.
(TIF)

Figure S2 **Sample data traces collected during Dynastat-based compression testing of mouse lenses.** (A) Compressive strain-time and (B) stress-time traces for a wild-type lens are shown. Strains of 0%, 5%, 10%, 15%, 20%, 15%, 10%, 5%, and 0% of the lens thickness were imposed to construct loading and unloading stress-strain curves (hysteresis loops). Each ramp change in strain was followed by a 5-min hold to permit stress relaxation.
(TIF)

Figure S3 **Deletion of Tmod1 and/or CP49 does not impact the size or shape of mouse lenses.** (A) Axial diameter, (B) equatorial diameter, (C) aspect ratio, and (D) volume were all unchanged in 2-mo-old mouse lenses lacking Tmod1 and/or CP49, both before the start of coverslip-based compressive loading and after unloading all 10 coverslips, indicating no permanent plastic deformation induced by the coverslip-based compression procedure. (E) The diameter of the rigid lens nucleus was unchanged in lenses lacking Tmod1 and/or CP49. (F) The nucleus occupied ∼65% of the axial diameter of the lens, as reflected by the ratio of the nuclear/axial diameter. During coverslip-based compressive loading, the ratio of nuclear/axial diameter increased to ∼100% at maximum compressive loading of 10 coverslips. Error bars reflect mean±SEM of *n* = 8 lenses/genotype. **, *p*<0.01.
(TIF)

Figure S4 **Lenses do not exhibit hysteresis when subjected to Dynastat-based compressive testing, and this property is not affected by absence of Tmod1 or CP49.** Hysteresis loops from 2-mo-old (A) wild-type, (B), *CP49−/−*, and (C) *CP49−/−;Tmod1−/−* lenses reveal no elastic energy dissipation due to plastic deformation. Loading curves are identical to those in Fig. 6A and not significantly different from their corresponding unloading curves. Error bars reflect mean±SEM of *n* = 8 lenses/genotype.
(TIF)
