# Supplementary figures and images for: Tmod1 and CP49 Synergize to Control the Fiber Cell Geometry, Transparency, and Mechanical Stiffness of the Mouse Lens
Source: PLoS One. 2012 Nov 7;7(11):e48734. doi: 10.1371/journal.pone.0048734 (PMC3492431; doi:10.1371/journal.pone.0048734)

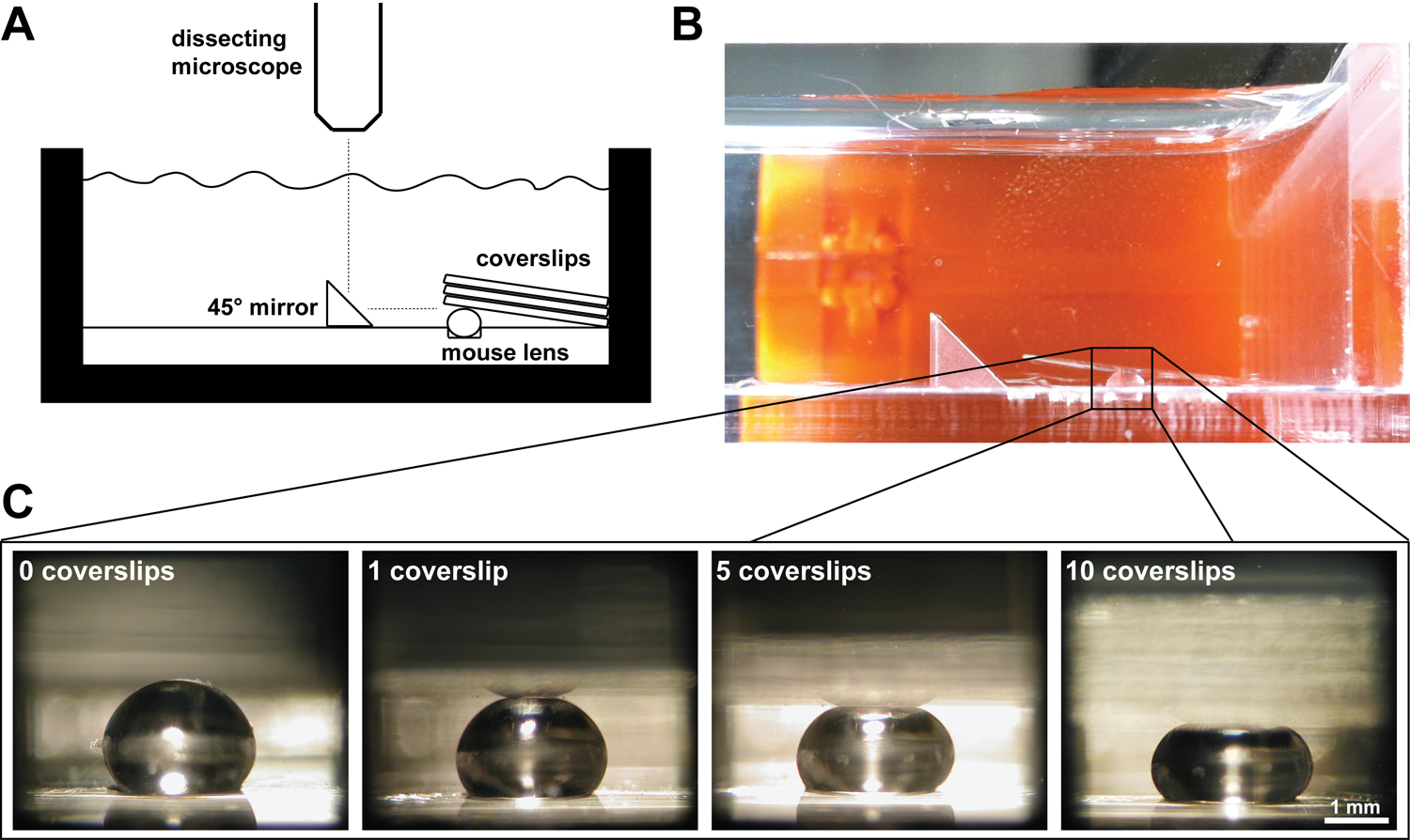

Supplement: Figure S1 — Experimental setup for coverslip-based compression testing of mouse lenses. (A) Schematic and (B) photograph of the experimental setup depict a mouse lens in a 200-µm-deep divot in an acrylic chamber filled with DPBS. To acquire sagittal images using a digital camera mounted on a dissecting microscope, a reflection of the lens was viewed through a 45° mirror. Compression was applied by lining up 10 coverslips along the edge of the chamber and carefully lowering them onto the lens, one at a time. Application of each coverslip was followed by 2 min of stress relaxation. (C) Photographs of sagittal views of lenses compressed by successively increasing numbers of coverslips provided the raw data for measuring axial and equatorial diameters and calculating axial and equatorial strains during coverslip-based compression testing. Axial diameters were corrected for the depth of the divot, which obscured 200 µm of the lens thickness. (TIF) [file pone.0048734.s001.tif]

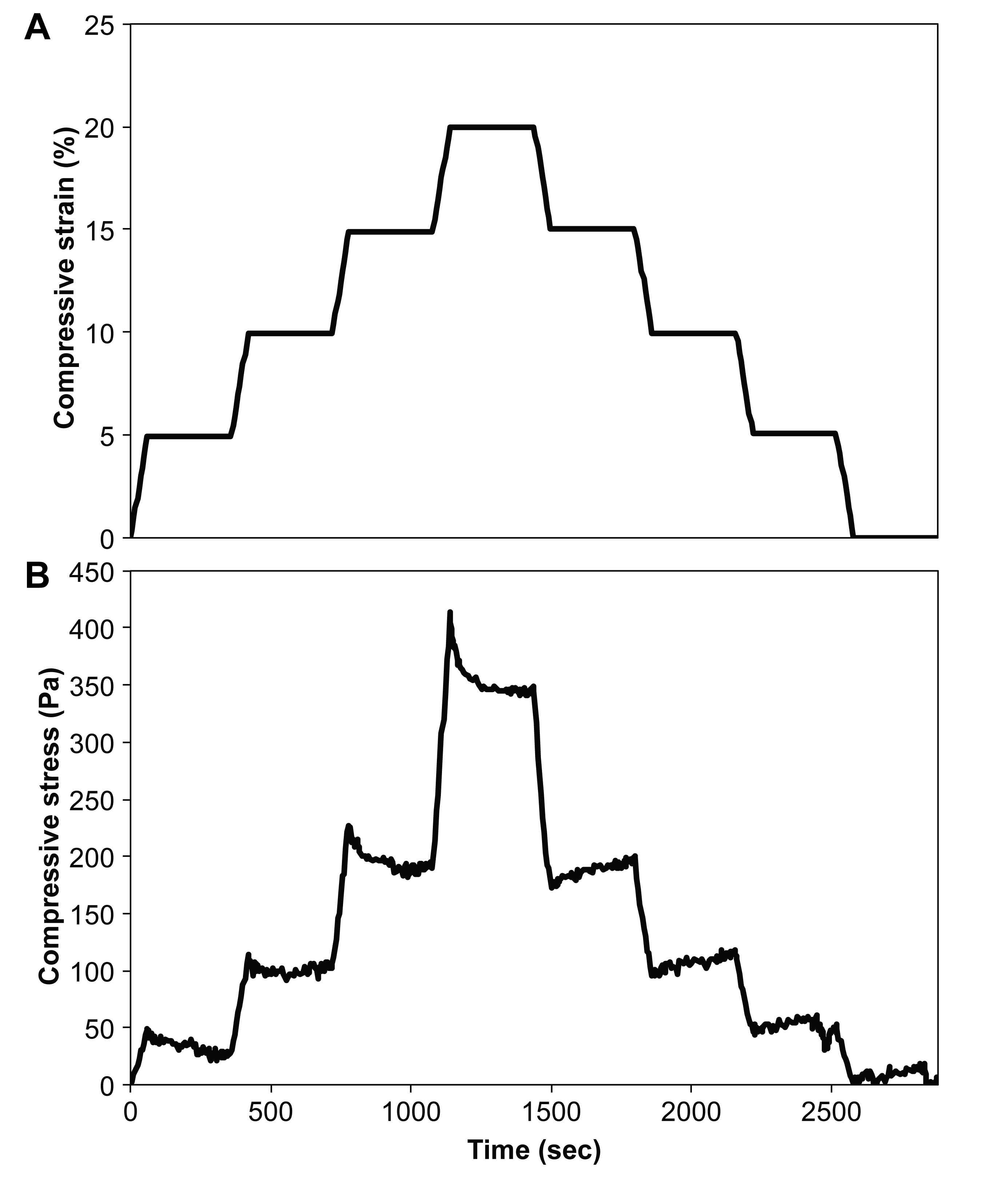

Supplement: Figure S2 — Sample data traces collected during Dynastat-based compression testing of mouse lenses. (A) Compressive strain-time and (B) stress-time traces for a wild-type lens are shown. Strains of 0%, 5%, 10%, 15%, 20%, 15%, 10%, 5%, and 0% of the lens thickness were imposed to construct loading and unloading stress-strain curves (hysteresis loops). Each ramp change in strain was followed by a 5-min hold to permit stress relaxation. (TIF) [file pone.0048734.s002.tif]

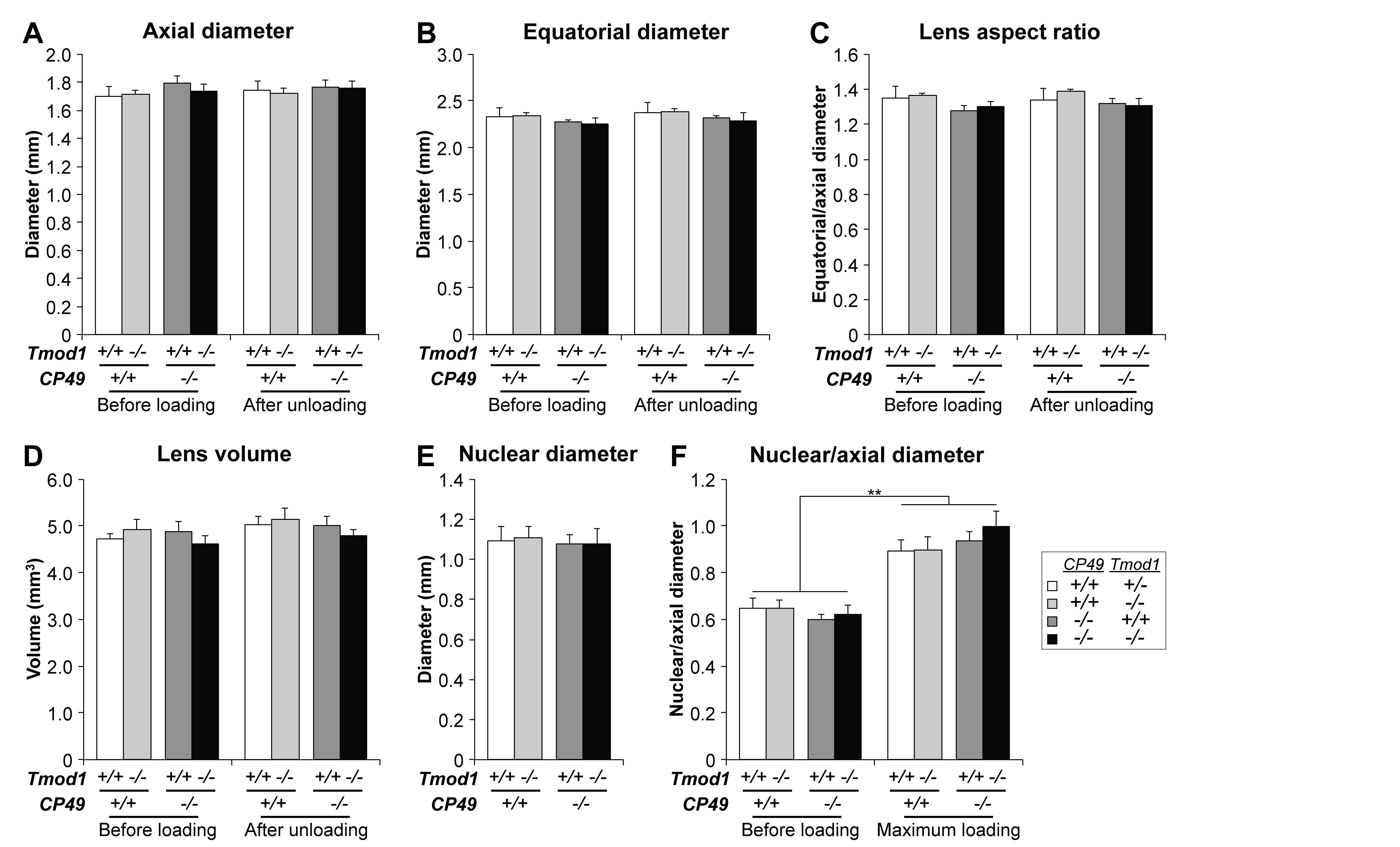

Supplement: Figure S3 — Deletion of Tmod1 and/or CP49 does not impact the size or shape of mouse lenses. (A) Axial diameter, (B) equatorial diameter, (C) aspect ratio, and (D) volume were all unchanged in 2-mo-old mouse lenses lacking Tmod1 and/or CP49, both before the start of coverslip-based compressive loading and after unloading all 10 coverslips, indicating no permanent plastic deformation induced by the coverslip-based compression procedure. (E) The diameter of the rigid lens nucleus was unchanged in lenses lacking Tmod1 and/or CP49. (F) The nucleus occupied ∼65% of the axial diameter of the lens, as reflected by the ratio of the nuclear/axial diameter. During coverslip-based compressive loading, the ratio of nuclear/axial diameter increased to ∼100% at maximum compressive loading of 10 coverslips. Error bars reflect mean±SEM of n = 8 lenses/genotype. **, p<0.01. (TIF) [file pone.0048734.s003.tif]

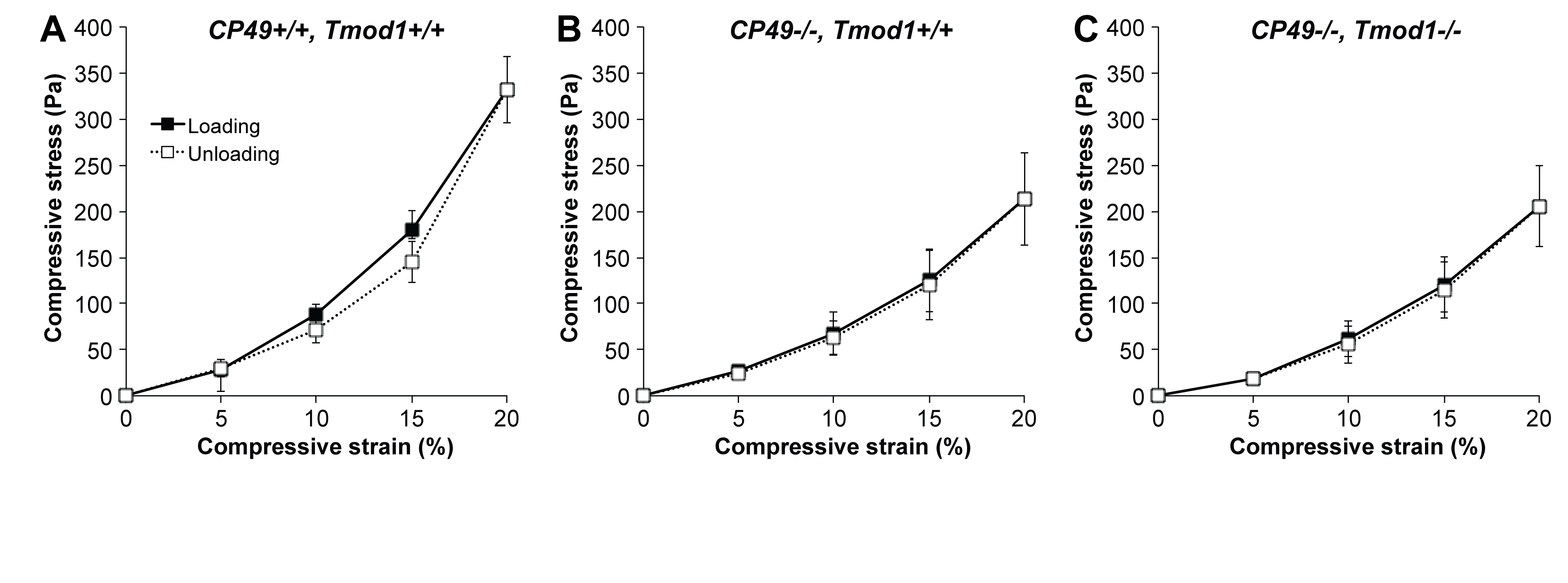

Supplement: Figure S4 — Lenses do not exhibit hysteresis when subjected to Dynastat-based compressive testing, and this property is not affected by absence of Tmod1 or CP49. Hysteresis loops from 2-mo-old (A) wild-type, (B), CP49−/−, and (C) CP49−/−;Tmod1−/− lenses reveal no elastic energy dissipation due to plastic deformation. Loading curves are identical to those in Fig. 6A and not significantly different from their corresponding unloading curves. Error bars reflect mean±SEM of n = 8 lenses/genotype. (TIF) [file pone.0048734.s004.tif]
